# Extrachromosomal Circular DNA as a Cancer Biomarker: From Diagnosis to Treatment

**DOI:** 10.1002/cai2.70026

**Published:** 2025-09-08

**Authors:** Hexin Li, Jiahui Cai, Gang Zhao, Lihui Zou

**Affiliations:** ^1^ Clinical Biobank, Beijing Hospital, National Center of Gerontology, National Health Commission, Institute of Geriatric Medicine Chinese Academy of Medical Sciences Beijing China; ^2^ Department of General Surgery Beijing Hospital Beijing China; ^3^ National Center of Gerontology, Institute of Geriatric Medicine Chinese Academy of Medical Sciences Beijing China; ^4^ The Key Laboratory of Geriatrics, Beijing Institute of Geriatrics, Institute of Geriatric Medicine, Chinese Academy of Medical Sciences Beijing Hospital/National Center of Gerontology of National Health Commission Beijing China

**Keywords:** biomarker, cancer, diagnosis, extrachromosomal circular DNA, treatment

## Abstract

Extrachromosomal circular DNA (eccDNA) is an emerging class of genetic material that exists outside of the chromosomal genome. These circular DNA molecules are gaining increasing attention as important biomarkers in various cancers because of their roles in gene amplification, genetic heterogeneity, and drug resistance. In this review, we explore in depth the impacts of eccDNAs on cancer biology, their potential to predict treatment sensitivity and resistance, and their involvement in the development of new anticancer therapies. eccDNAs can be used as biomarkers for various tumor types to help diagnose and predict prognosis.

## Graphical abstract

Extrachromosomal circular DNA (eccDNA) is an emerging class of genetic material that exists outside the chromosomal genome. These circular DNA molecules are gaining increasing attention as important biomarkers in cancer research due to their roles in gene amplification, genetic heterogeneity, and drug resistance. This review explores in depth its impact on cancer biological behavior, its potential to predict treatment sensitivity and resistance, and its role in the development of new therapies. eccDNA can be used as a biomarker for various tumors to help diagnose and predict prognosis.

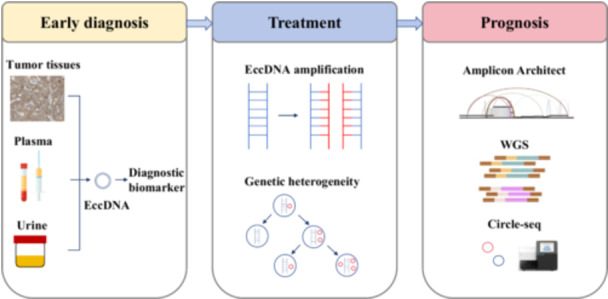

**Abbreviations**
eccDNA: extrachromosomal circular DNA
MEP1A: meprin A subunit alpha
WGS: whole‐genome sequencing

## 1 Background

Cancer remains one of the leading causes of death worldwide, making early detection and monitoring essential for improving patient outcomes [1]. With the increasing use of molecular diagnostics, the cancer treatment landscape is also evolving, which supports the development of personalized therapeutic intervention methods. Extrachromosomal circular DNA (eccDNA), a form of circular DNA that exists independently of chromosomes, is gaining recognition for its various roles in cancer. eccDNA is involved in gene amplification, genetic instability, and the development of drug resistance, all of which contribute to tumor progression and treatment failure [2].

As an emerging biomarker, eccDNA has shown promise in tumor diagnosis, treatment strategies, tumor behavior prediction, molecular subtyping, and understanding treatment sensitivity and drug resistance. In this review, we explore the comprehensive role of eccDNA in cancer and its huge potential in clinical oncology, from diagnostic strategies to novel therapeutic applications.

Here, we comprehensively summarize how eccDNA serves as a tumor diagnostic biomarker and its involvement in identifying new therapeutic drugs and strategies. We clarify its role in predicting tumor biological behavior, molecular typing, and treatment sensitivity/resistance. This detailed and comprehensive summary is more thorough than other recently published reviews in this field.

## 2 The Formation and Characteristics of eccDNA

**Figure 1:**
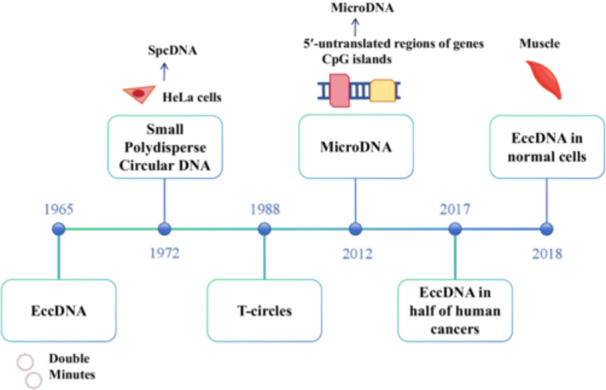
Timeline of the discoveries and research advancements related to extrachromosomal circular DNA (eccDNA). eccDNA was first observed in 1965 by Hotta and Bassel, after which multiple eccDNA categories, such as small polydispersed DNA (spcDNA), telomeric circles (t‐circles), and microDNA, have been identified in different species. Initially, eccDNA was believed to exist solely in tumor cells. However, in 2018, eccDNA molecules were found in normal cells.

**Figure 2:**
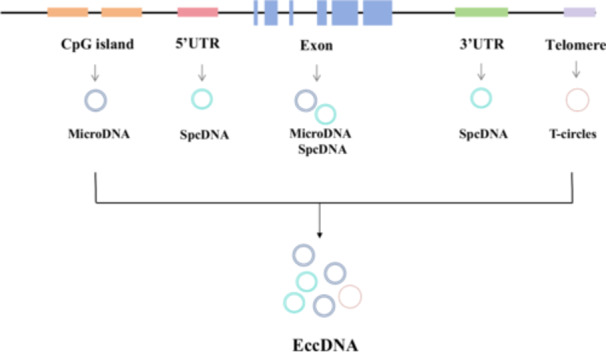
The origins of extrachromosomal circular DNA (eccDNA). UTR: Untranslated region.

Although eccDNAs originate from chromosomes, these closed‐circle DNA molecules exist independently of traditional chromosomal DNA after they are formed [3]. eccDNA was first observed in 1965 by Hotta and Bassel, who named it double minutes. eccDNA molecules have since been found in various organisms [2] (Figure 1). These circular DNA structures can vary in size from small fragments to large molecules and are classified into different types by their size and origin: small polydispersed DNA (spcDNA) (100 bp to 10 kb), telomeric circles (t‐circles) (multiples of 738 bp), microDNA (100–400 bp), and extrachromosomal DNA (ecDNA) (millions of bp) [4] (Table 1). spcDNA was first identified in HeLa cells in 1972 [7], while t‐circles have been studied since 1988 [8] and are known to play a key role in maintaining genomic stability. In 2012, Shibata et al. [9] identified microDNAs ranging from 200 to 400 bp as a new type of eccDNA. MicroDNA is primarily enriched in the 5′‐untranslated regions of genes, exons, and CpG islands. eccDNA can also harbor amplified genetic regions that might otherwise be overlooked in conventional genomic sequencing, making them particularly useful for detecting gene amplifications that contribute to cancer progression. The first systematic review of the discoveries of eccDNA in normal and tumor cells was published in 2018 [10] (Figure 2).

**Table 1:** Classification of extrachromosomal circular DNA (eccDNA).

Type	Size	Characteristic	Function	References
Small polydispersed DNA (spcDNA)	100 bp to 10 kb	Originates from repetitive regions in the genome	Involved in genomic instability	[2]
MicroDNA	200 bp to 400 bp	Enriched in the 5′‐untranslated regions of genes, exons, and CpG islands	Encodes functional small regulatory RNAs	[4]
Telomeric circles (t‐circles)	Multiples of 738 bp	Consist only of telomeric repeats	Circular DNA involved in the alternative lengthening and rapid deletion of telomeres	[5]
Extrachromosomal DNA (ecDNA)	Millions of bp	Lacks centromeres and segregates randomly or asymmetrically during cell division	Promotes tumor heterogeneity, tumor evolution, and drug resistance	[6]

eccDNA formation involves various mechanisms, such as DNA breakage, circularization, and gene amplification, leading to the enrichment of certain oncogenes in these extrachromosomal molecules. The presence of eccDNA is a hallmark of many cancer types and contributes to genetic diversity and tumor progression.

## 3 The Functional Mechanism of eccDNA in Cancer

Recent investigations have revealed that eccDNA plays an important role in cancer biology. Whole‐genome sequencing (WGS) and whole‐exome sequencing (WES) have shown great promise in accurately detecting and quantifying eccDNAs in cancer samples, making it feasible to track the large amounts of these molecules produced during tumorigenesis. Using next‐generation sequencing (NGS) methods, the latest eccDNA‐related discoveries in cancer have reignited significant interest. Because of technological advances in the bioinformatics field, eccDNA has gained further recognition as a novel molecule type, with many of its unique characteristics and functions now being uncovered [4, 11]. Notably, eccDNA molecules can carry genetic elements, such as oncogenes, regulatory sequences, or repetitive DNA sequences, highlighting their potential role in genetic diversity and tumor progression. These circular DNA molecules are formed through various mechanisms, including DNA replication, double‐strand break repair, and DNA damage response processes, further underscoring their involvement in cellular functions and genome instability.

eccDNAs are commonly found in both normal and cancer cells. Compared with non‐cancer tissues, cancer tissues often display eccDNA enrichment that contains a variety of genes involved in tumorigenesis, including oncogenes, as shown by Luo et al. [12]. From eccDNA detection technology advancements, recent investigations have revealed that eccDNAs can serve as multifunctional molecules when subjected to exogenous and endogenous stimuli in different biogenesis theories. Importantly, complete genes can be encoded within eccDNA molecules, supporting the ability to perform most of their functions, such as aging, oncogene amplification, genetic heterogeneity, drug resistance, immune‐related functions, and transcriptional regulation [2].

### 3.1 Oncogene Amplification

Studies have suggested that eccDNA amplification occurs frequently in most cancer types [13]. eccDNAs often carry amplified oncogenes that drive tumor progression, directly leading to an elevated DNA copy number of the oncogenes located on the extrachromosomal element.

### 3.2 Genetic Heterogeneity

More importantly, amplification of oncogenes via eccDNA contributes to the genetic heterogeneity of tumors, providing a survival advantage in response to environmental stressors, such as chemotherapy. Specifically, eccDNA is a stable, independent genetic entity that lacks centromeres, leading to its random or asymmetric segregation during cell division. These eccDNAs can bind to mitotic or meiotic chromosomes and become inherited by the next generation of cells, resulting in gene copy number changes that accumulate much more rapidly than with chromosomal DNA [14]. As a result, the initial eccDNA diversity was formed. More importantly, to ensure consistent eccDNA levels within the same population, eccDNA actively promotes transcription during mitosis, which differs from chromosomal gene transcription. Additionally, when the eccDNA transcription process is not fully completed, gene replication and mitosis are inhibited, ensuring that the eccDNA population can be inherited as a whole by the progeny cells. This mechanism allows advantageous traits to always be transmitted, which also supports the survival and progression of cancer cells [15]. The genetic heterogeneity leads to different phenotypes, such as tumor cells obtaining a proliferation advantage. This heterogeneity complicates treatment strategies and drives tumor evolution [13].

### 3.3 Transcriptional Regulation

Another eccDNA mechanism is that these molecules can drive RNA expression through transcription factor interactions [16]. Depending on the region of origin, eccDNAs can be classified into four categories: intergenic eccDNA, exon eccDNA, intron eccDNA, and intron–exon eccDNA. Moreover, in one study, intergenic eccDNAs and exon eccDNAs accounted for 58.6% and 38.2% of the total eccDNAs, respectively [17]. Importantly, eccDNAs can accordingly be transcribed into all defined transcript types, including mRNAs, microRNAs (miRNAs), long noncoding RNAs, and circular RNAs [3].

Recent studies have shown that chromatin remodeling complexes play a crucial role in the formation and regulation of eccDNA. A study by Crosetto et al. revealed that eccDNAs are preferentially produced from chromatin regions enriched in H3K9me3 and H3K27me3 histone marks [18], suggesting that eccDNA generation is closely linked to specific epigenetic marks on chromatin. Transcription factors can also bind to eccDNA molecules to regulate gene expression, often enhancing oncogene expression patterns. This interaction plays a significant role in cancer and other diseases. For example, Chen et al. found that eccDNA can promote prostate cancer progression through the FAM84B/CDKN1B/MYC/WWP1 axis [19].

### 3.4 Drug Resistance

In addition, eccDNAs promote the development of drug resistance by amplifying genes involved in the resistance pathway. eccDNA inheritance by daughter cells, rather than telomere involvement, leads to the rapid proliferation of drug‐resistant clones.

Overall, the functional mechanisms of eccDNA include oncogene amplification, genetic heterogeneity, transcriptional regulation, and participation in drug resistance, with functional variation promoted through genetic diversity.

## 4 Technological Advances in eccDNA Detection

The ability to accurately detect eccDNA is vital for its clinical application. Traditional techniques, such as NGS and fluorescence in situ hybridization (FISH), have been used to explore eccDNA sequence information, subcellular localization, and biological functions. However, these methods may not fully detect low‐copy eccDNAs, and bioinformatics analysis can struggle to distinguish eccDNA from other DNA types because of overlapping structural features.

Recent advancements in third‐generation sequencing technologies, such as long‐read sequencing, Circle‐seq, and single‐cell sequencing (SC‐Seq), have revolutionized eccDNA detection by providing higher resolution and deeper insights at the single‐base pair or single‐cell level [18]. The combination of long‐read sequencing and polymerase chain reaction‐free eccDNA purification techniques can overcome the sequencing limitations of traditional methods. Moreover, SC‐Seq is emerging as a dominant technology for investigating the roles of eccDNAs in cancers and other diseases [20]. Notably, scCircle‐seq is a powerful technique for mapping eccDNAs, enabling the exploration of their diversity and complexity at the single‐cell level. This scalable approach provides a means to analyze the intricate landscape of eccDNAs across various cell types and tissues, significantly enhancing the potential of using eccDNAs as cancer diagnostic biomarkers [18]. Moreover, bioinformatics tools, like Amplicon Architect, enable the identification of focal eccDNA amplifications directly from WGS data, making eccDNA detection more accurate and efficient. GCAP, another newly developed machine learning modeling tool, has been used to predict eccDNA amplification and its associated genes in tumor genomes [21].

**Figure 3:**
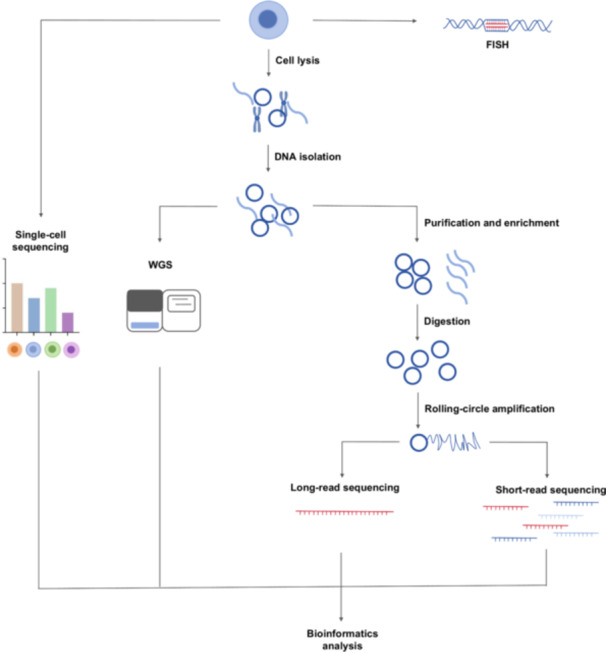
Flowchart for extrachromosomal circular DNA (eccDNA) investigations. The schematic provides comprehensive details of the tools and methods involved in eccDNA investigations. WGS: Whole‐genome sequencing.

In recent years, the development of bioinformatics tools and databases has greatly advanced eccDNA research and promoted significant progress in the field [22] (Figure 3). However, our understanding of the biological functions of eccDNAs remains incomplete, with many remaining gaps in knowledge [23]. The current detection technologies used to examine eccDNA face several limitations, including specificity issues and technical biases [24]. Amplification biases or library construction biases may lead to uneven detection of different eccDNA species. Extracting eccDNAs from tissues and plasma is problematic because of their low concentrations. Additionally, the presence of contaminating linear DNA has resulted in the requirement of a complex and time‐consuming procedure [25]. The current data remain limited, underscoring the need for ongoing technological innovation to further explore and harness the potential of eccDNAs in clinical applications.

## 5 eccDNA as a Cancer Biomarker

### 5.1 eccDNA as a Cancer Diagnostic Biomarker

By analyzing data from 14,778 patients with 39 tumor types, Mischel et al. found that 17.1% of these tumor samples had traces of eccDNA. In addition, the frequency of eccDNA varied by cancer type [15]. Currently, eccDNA has emerged as a promising diagnostic biomarker in cancer for its presence in both tumor tissues and body fluids, such as plasma, urine, and saliva. Traditional cancer diagnostic methods often rely on invasive tissue biopsies, which can be difficult and risky for patients. Because eccDNA is released into the bloodstream and other body fluids during tumor progression, it can serve as a noninvasive liquid biopsy tool, providing real‐time insights into tumor dynamics [12]. Therefore, eccDNA has the potential to become a diagnostic marker for early‐stage cancer, possibly being used in liquid biopsy to further monitor disease progression [26, 27]. Compared with other liquid biopsy components, eccDNA shows great promise for providing tumor‐specific insights. Exosomes and miRNAs offer multi‐dimensional data, but face challenges related to standardization and interpretation. Additionally, circulating tumor DNA is well‐established but may not be effective for all cancer types. eccDNAs serve as diagnostic biomarkers in many cancers. For example, in hepatocellular carcinoma and colorectal cancer, a small eccDNA originating from the meprin A subunit alpha (*MEP1A*) was validated in plasma samples. The findings confirmed that *MEP1A* plays a significant role in cancer diagnosis [12, 28, 29]. Additionally, the levels of a small eccDNA originating from myosin 18B (*MYO18B*) were found to be significantly elevated in cancer patient plasma samples. *MYO18B* is regarded as a potential tumor suppressor gene in various types of cancer. Moreover, the combination of small eccDNAs originating from *ETV6* and *ALK* showed high multi‐cancer diagnostic value in tissues [12]. In addition to being detectable in plasma, eccDNAs are also present in the bile of patients with perihilar cholangiocarcinoma. Notably, significantly higher levels of eccDNAs were detected in the bile than in the plasma [30].

### 5.2 eccDNA as a Predictor of Treatment Sensitivity and Resistance

eccDNA is crucially involved in helping to predict cancer treatment outcomes. One of the most significant roles of eccDNA in cancer is its contribution to therapeutic sensitivity and drug resistance. eccDNA‐derived oncogene amplification can influence the responses to chemotherapy, targeted therapies, and immunotherapy in several tumor types [31, 32]. Tumors with eccDNA amplification often exhibit greater resistance to treatment, which complicates therapeutic strategies. As a result, cancer treatment regimens often fail because of the presence of genetic alterations, including gene amplifications and mutations. Some studies have suggested that eccDNA amplification frequently occurs in most cancer types, but not in normal tissues [33]. Wu et al. analyzed gene expression patterns in the tumor transcriptome and found that the oncogenes encoding eccDNAs were among the most highly expressed genes, implying that an increase in copy number leads to higher levels of transcription. This characteristic allows eccDNA to replicate autonomously within cancer cells, driving the overexpression of key genes and promoting the growth and proliferation of tumor cells. For example, eccDNAs can carry certain highly expressed oncogenes, such as *MYC* and *ERBB2*, which are present in abundant copy numbers in different cancer cell lines [34]. This further leads to drug resistance or activation of various oncogenes. WGS analysis of different cancer types suggested that eccDNA amplification has increased the oncogene copy number in various cancers [35]. For example, *DHFR* gene amplification confers resistance to MTX in tumors by elevating the production of DHFR [6]. The presence of eccDNA harboring such amplifications can help predict whether a patient will respond to a given therapy or develop resistance over time. Additionally, different subclones within the same tumor can carry distinct eccDNA profiles because of the unequal division of eccDNAs. The resulting genetic heterogeneity can lead to differential responses to treatment [33, 36]. eccDNA transcription can temporarily inhibit the genome replication process, with Mischel et al. finding this mechanism to be a potential point of therapeutic intervention. Research has found that a molecule called pRPA2‐S33 can increase eccDNA transcription. At the same time, the inhibition of DNA replication triggers a signaling cascade that activates *CHK1*, preventing mitosis from occurring before DNA replication is complete. *CHK1* inhibitors have been found to effectively inhibit cancer growth in tumor cells containing eccDNA.

Overall, by tracking eccDNA in blood samples, clinicians may be able to predict how sensitive a tumor is to specific therapies, enabling more personalized treatment plans. Furthermore, studying eccDNA can help identify drug resistance mechanisms, providing insights into potential strategies to overcome this resistance and improve treatment efficacy.

### 5.3 The Role of eccDNA in Novel Cancer Therapeutics

As our understanding of eccDNA continues to deepen, so does its potential as a novel cancer therapeutic target. Targeting eccDNA, either the molecules directly or the pathways involved in their formation, could provide new therapeutic options, especially for tumors that exhibit high levels of eccDNA‐mediated gene amplification. Focusing on inhibiting eccDNA formation and maintenance could lead to the development of new targeted therapies, which may offer significant improvements in treatment effectiveness and help overcome resistance to conventional methods.

Identifying specific eccDNA‐derived oncogenes could lead to targeted therapies as potential strategies for eliminating eccDNA. Some studies have proposed that enhancing certain DNA repair or DNA damage responses might prevent eccDNA formation [37]. For example, alternative end‐joining (alt‐EJ) is crucial for eccDNA production. This process drives eccDNA biogenesis, as silencing any of the associated factors completely abolishes the formation of 1‐LTR eccDNA in HMS‐Beagle cells. This highlights the essential role of alt‐EJ in the formation of eccDNA [38]. Consequently, DNA‐dependent protein kinase inhibitors can affect these factors to physically eliminate or silence eccDNAs, potentially providing a novel strategy for controlling eccDNA‐related genomic instability or diseases.

Moreover, targeting eccDNA maintenance could offer new therapeutic possibilities. Recent research has suggested that cancer cells containing eccDNA molecules have unique vulnerabilities. Replication stress can result in replication disaster, a major cause of cell death [39]. Tumors with eccDNAs experience high replication stress from the presence of these extra‐chromosomal elements. Mischel et al. demonstrated that an experimental inhibitor (BBI‐2779, related to *CHK1* inhibition) could exploit the DNA copying difficulties in eccDNA‐positive cancer cells, causing catastrophic replication failure when combined with targeted therapy [40]. This approach led to dramatic tumor regression in preclinical models.

In addition, overcoming drug resistance by targeting eccDNA has emerged as an effective therapeutic strategy. Because eccDNAs often harbor genes that confer drug resistance, which enable tumors or pathogens to evade the effects of treatment. By targeting these circular DNA structures, therapeutic approaches aim to prevent or counteract these resistance mechanisms. This strategy holds significant potential, as it could not only block the function of resistance genes carried by eccDNA but also help to suppress tumor growth or pathogen spread. Such an approach may offer a novel way to combat multidrug resistance, enhancing the effectiveness of existing therapies. This therapeutic angle is especially promising in cases where traditional treatments fail due to acquired resistance.

### 5.4 eccDNA in Cancer Prognosis

eccDNA is also highly valuable for assessing cancer prognosis. Kumar et al. found that in nearly two‐thirds of patients with lung and ovarian tumors, cell‐free eccDNA was longer before tumor resection than after. These molecules have been detected in plasma and serum samples, indicating that eccDNAs are released into circulation from the tumor tissues [27]. Additional work confirmed that MEP1A plays a significant role in cancer prognosis [28]. Research has shown that the polycistronic miR‐17–92 locus carried by eccDNA is associated with poor hepatocellular carcinoma patient prognosis [41, 42]. Cen et al. found that lower expression levels of DNMT1circle10302690‐10302961 in both primary and metastatic tumors were associated with a worse prognosis of high‐grade serous ovarian cancer patients [43]. Additionally, eccDNA‐based molecular profiling analysis significantly improved the accuracy of identifying glioma, as well as assessing the prognosis and predicting recurrence in patients [44]. Other studies have demonstrated that eccDNA derived from *PLCG2* is highly expressed in lung cancer tissues and tends to be associated with poor prognosis [45]. Furthermore, extrachromosomal *MYCN* amplification has been linked to worse patient outcomes [46]. Moreover, in cholangiocarcinoma, the enrichment of eccDNAs in the WNT and MAPK pathways could be linked to a poor prognosis [30]. Another example is in medulloblastoma, where eccDNA levels could be used to predict dismal prognosis. Patients with eccDNAs had significantly lower overall survival and disease‐free survival rates than patients not carrying these eccDNAs [47]. Therefore, eccDNAs may serve not only as diagnostic markers but also as a prognostic analysis tool in cancers.

**Figure 4:**
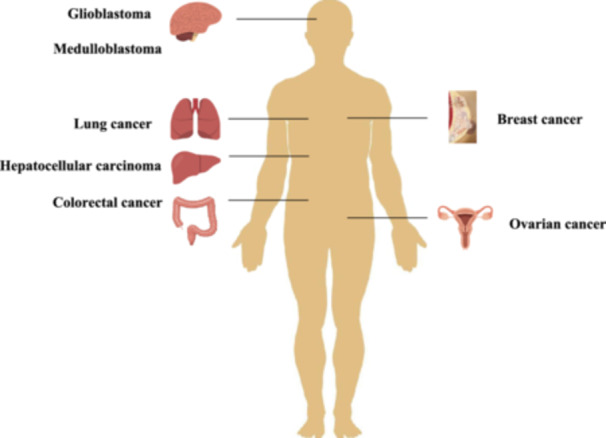
Functions of extrachromosomal circular DNA (eccDNA) in different cancer types. eccDNA plays functional diagnostic, therapeutic, and prognostic roles in a variety of cancers.

Existing studies have shown that patients with eccDNA‐based oncogene amplification have significantly shorter survival compared with those without eccDNA‐driven cancers [33]. Further investigations into eccDNA‐amplified oncogenes may provide more reliable cancer prognosis predictions. Exploring the behavioral specificity of eccDNA in different tumors could also help clarify its prognostic significance across various cancer types (Figure 4). The use of bioinformatics tools plays an important role in this. For example, amplicon reconstruction instruments, such as Amplicon Architect, support the identification of focal amplification and eccDNAs directly from WGS data. This tool can selectively identify eccDNAs through their biological characteristics, offering a powerful means to isolate and study oncogene‐amplified eccDNAs [36]. The combination of WGS and Amplicon Architect enables the precise selection of eccDNAs from chromosomal DNA, while Circle‐seq helps identify sequencing errors and improves the accuracy of eccDNA detection. Applying eccDNAs as prognostic biomarkers is becoming increasingly feasible, promising new avenues for personalized cancer treatment and prognostic evaluation.

## 6 Clinical Implications and Future Directions

With research advancements, the potential for eccDNA clinical applications has become increasingly evident. The detection of eccDNA offers a powerful tool for cancer diagnosis, monitoring treatment efficacy and predicting recurrence.

In the future, eccDNA could play a pivotal role in the following: (1) Liquid biopsy: noninvasive testing for eccDNAs in blood, urine, or saliva samples could revolutionize early cancer detection, allowing real‐time monitoring of tumor progression and treatment response; (2) Targeted therapies: by identifying specific eccDNA‐derived oncogenes, targeted therapies could be developed to inhibit eccDNA formation or functioning, potentially overcoming resistance to conventional treatments; and (3) Combination therapies: understanding the role of eccDNAs in drug resistance may lead to the development of novel combination therapies that target eccDNA‐related pathways. When integrated with radiation therapy and other chemotherapeutic agents, these therapies may enhance treatment efficacy, providing a more comprehensive and synergistic approach to cancer treatment [37].

In conclusion, eccDNA is a dynamic and multifaceted biomarker with significant potential in cancer diagnosis, prognosis, and treatment. For clinical translation, practical challenges include variability in eccDNA profiles between individuals, which may affect the reliability of diagnostics across diverse populations. Standardization of detection protocols is crucial to ensure consistent and reproducible results across different labs and settings. Additionally, regulatory hurdles must be navigated to ensure that eccDNA‐based diagnostics meet the safety, efficacy, and ethical standards for clinical use. Nowadays, in many countries and regions, new diagnostic technologies or biomarkers must undergo strict approval and validation processes before clinical application. Additionally, without sufficient incentives, companies often lack strong innovation capabilities, leading to therapies that offer even modest improvements in the benefit‐to‐risk ratio relative to existing treatments being increasingly prioritized for licensure [48]. However, eccDNA, as a relatively new type of biomarker, faces significant regulatory challenges because of limited clinical validation data. Therefore, with continued research and technological advancements, eccDNA‐based strategies may lead to more personalized and effective cancer therapies.

## 7 Conclusion

**Figure 5:**
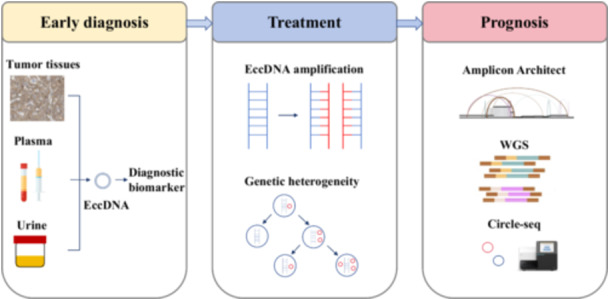
The role of extrachromosomal circular DNA (eccDNA) in cancer progression. WGS: Whole‐genome sequencing.

In oncology, the roles of eccDNAs in gene amplification, heterogeneity, tumor progression, and drug resistance highlight their potential as valuable tools for early diagnosis, prognosis, and personalized treatment strategies in various cancers (Figure 5). In this context, the screening of cancer‐specific or cancer‐associated eccDNAs further enables the discovery of novel biomarkers and promising therapeutic targets for cancer diagnostics and therapeutics [23]. However, for clinical implementation, substantial improvements must be made to the current sensitivity of eccDNA detection methods. This necessitates the adoption of advanced sequencing technologies and the development of more sensitive detection tools to obtain rapid and accurate results. It is critical to distinguish eccDNA from chromosomal DNA to avoid potential confounding. Moreover, technological advancements are pivotal for elucidating the mechanisms of eccDNA formation and its biological significance in cancer. Furthermore, progress in novel targeted technologies will be essential for advancing targeted therapies using eccDNA. In summary, research on eccDNA has provided valuable insights into the indispensable role of this genetic material in cancer progression. Moreover, as our understanding of eccDNA expands, further refined and innovative investigations in this field could significantly influence cancer diagnostic processes, treatment approaches, and patient outcomes, thereby offering promising strategies for inhibiting cancer progression.

## Author Contributions

**Hexin Li:** conceptualization (lead), resources (equal), supervision (equal), visualization (lead), writing – original draft (equal). **Jiahui Cai:** writing – original draft (lead), writing – review and editing (equal), investigation (lead), visualization (equal). **Gang Zhao:** funding acquisition (lead), project administration (lead), supervision (lead). **Lihui Zou:** writing – review and editing (lead), resources (equal), supervision (equal).

## Ethics Statement

The authors have nothing to report.

## Consent

The authors have nothing to report.

## Conflicts of Interest

The authors declare no conflicts of interest.

## Data Availability

Data sharing is not applicable to this article as no datasets were generated or analyzed during the current study.

## References

[1] M. Naghavi , K. L. Ong , A. Aali , et al., GBD 2021 Causes of Death Collaborators , “Global Burden of 288 Causes of Death and Life Expectancy Decomposition in 204 Countries and Territories and 811 Subnational Locations, 1990–2021: A Systematic Analysis for the Global Burden of Disease Study 2021,” Lancet 403, no. 10440 (2024): 2100–2132, 10.1016/S0140-6736(24)00367-2. 38582094 PMC11126520

[2] L. Yang , R. Jia , T. Ge , et al., “Extrachromosomal Circular DNA: Biogenesis, Structure, Functions and Diseases,” Signal Transduction and Targeted Therapy 7, no. 1 (2022): 342, 10.1038/s41392-022-01176-8. 36184613 PMC9527254

[3] X. Ling , Y. Han , J. Meng , et al., “Small Extrachromosomal Circular DNA (eccDNA): Major Functions in Evolution and Cancer,” Molecular Cancer 20, no. 1 (2021): 113, 10.1186/s12943-021-01413-8. 34479546 PMC8414719

[4] Z. Liao , W. Jiang , L. Ye , T. Li , X. Yu , and L. Liu , “Classification of Extrachromosomal Circular DNA With a Focus on the Role of Extrachromosomal DNA (ecDNA) in Tumor Heterogeneity and Progression,” Biochimica et Biophysica Acta (BBA)—Reviews on Cancer 1874, no. 1 (2020): 188392, 10.1016/j.bbcan.2020.188392. 32735964

[5] L. Tomaska , M. J. McEachern , and J. Nosek , “Alternatives to Telomerase: Keeping Linear Chromosomes via Telomeric Circles,” FEBS Letters 567, no. 1 (2004): 142–146, 10.1016/j.febslet.2004.04.058. 15165907

[6] K. M. Turner , V. Deshpande , D. Beyter , et al., “Extrachromosomal Oncogene Amplification Drives Tumour Evolution and Genetic Heterogeneity,” Nature 543, no. 7643 (2017): 122–125, 10.1038/nature21356. 28178237 PMC5334176

[7] C. A. Smith and J. Vinograd , “Small Polydisperse Circular DNA of HeLa Cells,” Journal of Molecular Biology 69, no. 2 (1972): 163–178, 10.1016/0022-2836(72)90222-7. 5070865

[8] B. Timmerman , M. Van Montagu , and P. Zambryski , “ *Vir*‐Induced Recombination in *Agrobacterium* ,” Journal of Molecular Biology 203, no. 2 (1988): 373–384, 10.1016/0022-2836(88)90005-8. 3199438

[9] Y. Shibata , P. Kumar , R. Layer , et al., “Extrachromosomal MicroDNAs and Chromosomal Microdeletions in Normal Tissues,” Science 336, no. 6077 (2012): 82–86, 10.1126/science.1213307. 22403181 PMC3703515

[10] T. Paulsen , P. Kumar , M. M. Koseoglu , and A. Dutta , “Discoveries of Extrachromosomal Circles of DNA in Normal and Tumor Cells,” Trends in Genetics 34, no. 4 (2018): 270–278, 10.1016/j.tig.2017.12.010. 29329720 PMC5881399

[11] P. Kumar , S. Kiran , S. Saha , et al., “ATAC‐Seq Identifies Thousands of Extrachromosomal Circular DNA in Cancer and Cell Lines,” Science Advances 6, no. 20 (2020): eaba2489, 10.1126/sciadv.aba2489. 32440553 PMC7228742

[12] X. Luo , L. Zhang , J. Cui , et al., “Small Extrachromosomal Circular DNAs as Biomarkers for Multi‐Cancer Diagnosis and Monitoring,” Clinical and Translational Medicine 13, no. 9 (2023): e1393, 10.1002/ctm2.1393. 37649244 PMC10468585

[13] R. G. W. Verhaak , V. Bafna , and P. S. Mischel , “Extrachromosomal Oncogene Amplification in Tumour Pathogenesis and Evolution,” Nature Reviews Cancer 19, no. 5 (2019): 283–288, 10.1038/s41568-019-0128-6. 30872802 PMC7168519

[14] D.‐H. Koo , R. Sathishraj , B. Friebe , and B. S. Gill , “Deciphering the Mechanism of Glyphosate Resistance in *Amaranthus palmeri* by Cytogenomics,” Cytogenetic and Genome Research 161, no. 12 (2021): 578–584, 10.1159/000521409. 35021177

[15] C. Bailey , O. Pich , K. Thol , et al., “Origins and Impact of Extrachromosomal DNA,” Nature 635, no. 8037 (2024): 193–200, 10.1038/s41586-024-08107-3. 39506150 PMC11540846

[16] R. M. Hull , M. King , G. Pizza , F. Krueger , X. Vergara , and J. Houseley , “Transcription‐Induced Formation of Extrachromosomal DNA During Yeast Ageing,” PLoS Biology 17, no. 12 (2019): e3000471, 10.1371/journal.pbio.3000471. 31794573 PMC6890164

[17] M. Zhu , X. Tong , Q. Qiu , et al., “Identification and Characterization of Extrachromosomal Circular DNA in the Silk Gland of *Bombyx mori* ,” Insect Science 30, no. 6 (2023): 1565–1578, 10.1111/1744-7917.13191. 36826848

[18] J. P. Chen , C. Diekmann , H. Wu , et al., “ScCircle‐Seq Unveils the Diversity and Complexity of Extrachromosomal Circular DNAs in Single Cells,” Nature Communications 15, no. 1 (2024): 1768, 10.1038/s41467-024-45972-y. PMC10897160 38409079

[19] W. Jin , Z. Xu , Y. Song , and F. Chen , “Extrachromosomal Circular DNA Promotes Prostate Cancer Progression Through the FAM84B/CDKN1B/MYC/WWP1 Axis,” Cellular & Molecular Biology Letters 29, no. 1 (2024): 103, 10.1186/s11658-024-00616-3. 38997648 PMC11245840

[20] H. van den Bos , B. Bakker , D. Spierings , P. M. Lansdorp , and F. Foijer , “Single‐Cell Sequencing to Quantify Genomic Integrity in Cancer,” International Journal of Biochemistry & Cell Biology 94 (2018): 146–150, 10.1016/j.biocel.2017.09.016. 28951245

[21] S. Wang , C.‐Y. Wu , M.‐M. He , et al., “Machine Learning‐Based Extrachromosomal DNA Identification in Large‐Scale Cohorts Reveals Its Clinical Implications in Cancer,” Nature Communications 15, no. 1 (2024): 1515, 10.1038/s41467-024-45479-6. PMC10876971 38373991

[22] S. Wu , K. M. Turner , N. Nguyen , et al., “Circular ecDNA Promotes Accessible Chromatin and High Oncogene Expression,” Nature 575, no. 7784 (2019): 699–703, 10.1038/s41586-019-1763-5. 31748743 PMC7094777

[23] M. Wang , X. Chen , F. Yu , H. Ding , Y. Zhang , and K. Wang , “Extrachromosomal Circular DNAs: Origin, Formation and Emerging Function in Cancer,” International Journal of Biological Sciences 17, no. 4 (2021): 1010–1025, 10.7150/ijbs.54614. 33867825 PMC8040306

[24] M. A. Atieh , N. H. Alsabeeha , A. G. Payne , S. Ali , C. M. Faggion , and M. Esposito , “Interventions for Replacing Missing Teeth: Alveolar Ridge Preservation Techniques for Dental Implant Site Development,” Cochrane Database of Systematic Reviews 4, no. 4 (2021): CD010176, 10.1002/14651858.cd010176.pub2. 33899930 PMC8092674

[25] E. Zole , G. Sathyanarayanan , B. Regenberg , and J. P. Kutter , “Microfluidic Isolation of Extrachromosomal Circular DNA Through Selective Digestion of Plasmids and Linear DNA Using Immobilized Nucleases,” Lab on a Chip 24, no. 12 (2024): 3101–3111, 10.1039/D3LC01028G. 38752699

[26] J. B. Noer , O. K. Hørsdal , X. Xiang , Y. Luo , and B. Regenberg , “Extrachromosomal Circular DNA in Cancer: History, Current Knowledge, and Methods,” Trends in Genetics 38, no. 7 (2022): 766–781, 10.1016/j.tig.2022.02.007. 35277298

[27] P. Kumar , L. W. Dillon , Y. Shibata , A. A. Jazaeri , D. R. Jones , and A. Dutta , “Normal and Cancerous Tissues Release Extrachromosomal Circular DNA (eccDNA) Into the Circulation,” Molecular Cancer Research 15, no. 9 (2017): 1197–1205, 10.1158/1541-7786.mcr-17-0095. 28550083 PMC5581709

[28] H.‐Y. OuYang , J. Xu , J. Luo , et al., “MEP1A Contributes to Tumor Progression and Predicts Poor Clinical Outcome in Human Hepatocellular Carcinoma,” Hepatology 63, no. 4 (2016): 1227–1239, 10.1002/hep.28397. 26660154

[29] X. Wang , J. Chen , J. Wang , et al., “Metalloproteases Meprin‐ɑ (MEP1A) Is a Prognostic Biomarker and Promotes Proliferation and Invasion of Colorectal Cancer,” BMC Cancer 16 (2016): 383, 10.1186/s12885-016-2460-5. 27378469 PMC4932728

[30] S. Fu , Y. Dai , P. Zhang , et al., “Extrachromosomal Circular DNA (eccDNA) Characteristics in the Bile and Plasma of Advanced Perihilar Cholangiocarcinoma Patients and the Construction of an EccDNA‐Related Gene Prognosis Model,” Frontiers in Cell and Developmental Biology 12 (2024): 1379435, 10.3389/fcell.2024.1379435. 38903532 PMC11187006

[31] X.‐K. Zhao , P. Xing , X. Song , et al., “Focal Amplifications Are Associated With Chromothripsis Events and Diverse Prognoses in Gastric Cardia Adenocarcinoma,” Nature Communications 12, no. 1 (2021): 6489, 10.1038/s41467-021-26745-3. PMC8586158 34764264

[32] O. Shoshani , S. F. Brunner , R. Yaeger , et al., “Chromothripsis Drives the Evolution of Gene Amplification in Cancer,” Nature 591, no. 7848 (2021): 137–141, 10.1038/s41586-020-03064-z. 33361815 PMC7933129

[33] H. Kim , N.‐P. Nguyen , K. Turner , et al., “Extrachromosomal DNA Is Associated With Oncogene Amplification and Poor Outcome Across Multiple Cancers,” Nature Genetics 52, no. 9 (2020): 891–897, 10.1038/s41588-020-0678-2. 32807987 PMC7484012

[34] J. P. Chen , C. Diekmann , H. Wu , et al., “Author Correction: ScCircle‐Seq Unveils the Diversity and Complexity of Extrachromosomal Circular DNAs in Single Cells,” Nature Communications 15, no. 1 (2024): 3390, 10.1038/s41467-024-45972-y. PMC11035616 38649373

[35] M. Karami Fath , M. Akbari Oryani , A. Ramezani , et al., “Extra Chromosomal DNA in Different Cancers: Individual Genome With Important Biological Functions,” Critical Reviews in Oncology/Hematology 166 (2021): 103477, 10.1016/j.critrevonc.2021.103477. 34534658

[36] R. Li , Y. Wang , J. Li , and X. Zhou , “Extrachromosomal Circular DNA (eccDNA): An Emerging Star in Cancer,” Biomarker Research 10, no. 1 (2022): 53, 10.1186/s40364-022-00399-9. 35883211 PMC9327165

[37] Q. Huang , S. Zhang , G. Wang , and J. Han , “Insight on EcDNA‐Mediated Tumorigenesis and Drug Resistance,” Heliyon 10, no. 6 (2024): e27733, 10.1016/j.heliyon.2024.e27733. 38545177 PMC10966608

[38] F. Yang , W. Su , O. W. Chung , et al., “Retrotransposons Hijack Alt‐EJ for DNA Replication and eccDNA Biogenesis,” Nature 620, no. 7972 (2023): 218–225, 10.1038/s41586-023-06327-7. 37438532 PMC10691919

[39] M. K. Zeman and K. A. Cimprich , “Causes and Consequences of Replication Stress,” Nature Cell Biology 16, no. 1 (2014): 2–9, 10.1038/ncb2897. 24366029 PMC4354890

[40] J. Tang , N. E. Weiser , G. Wang , et al., “Enhancing Transcription–Replication Conflict Targets EcDNA‐Positive Cancers,” Nature 635, no. 8037 (2024): 210–218, 10.1038/s41586-024-07802-5. 39506153 PMC11540844

[41] O. Andrisani , “Two Important Players in Poor‐Prognosis Hepatocellular Carcinoma: Extrachromosomal Circular DNA (eccDNA) and Its Passenger, the Oncogenic miR‐17~92 Locus,” Hepatology 79, no. 1 (2024): 6–8, 10.1097/hep.0000000000000453. 37183875 PMC11980994

[42] S. Zou , S. Chen , G. Rao , et al., “Extrachromosomal Circular miR‐17–92 Amplicon Promotes HCC,” Hepatology 79, no. 1 (2024): 79–95, 10.1097/hep.0000000000000435. 37125628

[43] Y. Cen , Y. Fang , Y. Ren , S. Hong , W. Lu , and J. Xu , “Global Characterization of Extrachromosomal Circular DNAs in Advanced High Grade Serous Ovarian Cancer,” Cell Death & Disease 13, no. 4 (2022): 342, 10.1038/s41419-022-04807-8. 35418185 PMC9007969

[44] Z. Li , B. Wang , H. Liang , Y. Li , Z. Zhang , and L. Han , “A Three‐Stage eccDNA Based Molecular Profiling Significantly Improves the Identification, Prognosis Assessment and Recurrence Prediction Accuracy in Patients With Glioma,” Cancer Letters 574 (2023): 216369, 10.1016/j.canlet.2023.216369. 37640198

[45] Y. Yang , Y. Yang , H. Huang , et al., “PLCG2 Can Exist in eccDNA and Contribute to the Metastasis of Non‐Small Cell Lung Cancer by Regulating Mitochondrial Respiration,” Cell Death & Disease 14, no. 4 (2023): 257, 10.1038/s41419-023-05755-7. 37031207 PMC10082821

[46] R. P. Koche , E. Rodriguez‐Fos , K. Helmsauer , et al., “Extrachromosomal Circular DNA Drives Oncogenic Genome Remodeling in Neuroblastoma,” Nature Genetics 52, no. 1 (2020): 29–34, 10.1038/s41588-019-0547-z. 31844324 PMC7008131

[47] O. S. Chapman , J. Luebeck , S. Sridhar , et al., “Circular Extrachromosomal DNA Promotes Tumor Heterogeneity in High‐Risk Medulloblastoma,” Nature Genetics 55, no. 12 (2023): 2189–2199, 10.1038/s41588-023-01551-3. 37945900 PMC10703696

[48] B. Jonsson and J. Bergh , “Hurdles in Anticancer Drug Development From a Regulatory Perspective,” Nature Reviews Clinical Oncology 9, no. 4 (2012): 236–243, 10.1038/nrclinonc.2012.14. 22349015

